# Supplementary material for: Leprosy perceptions and knowledge in endemic districts in India and Indonesia: Differences and commonalities
Source: PLoS Negl Trop Dis. 2021 Jan 21;15(1):e0009031. doi: 10.1371/journal.pntd.0009031 (PMC7853455; doi:10.1371/journal.pntd.0009031)
Supplement: S1 STROBE Checklist — (DOC) [file pntd.0009031.s001.doc]

STROBE Statement—Checklist of items that should be included in reports of ***cross-sectional studies***

**“Leprosy perceptions and knowledge in endemic districts in India and Indonesia: differences and commonalities”**

|  | Item No | Recommendation |
| --- | --- | --- |
| **Title and abstract** | 1 | (*a*) Indicate the study’s design with a commonly used term in the title or the abstract Abstract, paragraph 2 (under heading ‘methods’) |
|  |  | (*b*) Provide in the abstract an informative and balanced summary of what was done and what was found Abstract, paragraphs 2 and 3 |
| Introduction | | |
| Background/rationale | 2 | Explain the scientific background and rationale for the investigation being reported Introduction, all paragraphs; paragraphs 3 and 5 in particular |
| Objectives | 3 | State specific objectives, including any prespecified hypotheses Introduction, final paragraph |
| Methods | | |
| Study design | 4 | Present key elements of study design early in the paper Methods, section ‘study design’ |
| Setting | 5 | Describe the setting, locations, and relevant dates, including periods of recruitment, exposure, follow-up, and data collection Methods, section ‘study site’ |
| Participants | 6 | (*a*) Give the eligibility criteria, and the sources and methods of selection of participants Methods, sections ‘eligibility criteria’ and ‘sampling methods’ |
| Variables | 7 | Clearly define all outcomes, exposures, predictors, potential confounders, and effect modifiers. Give diagnostic criteria, if applicable Methods, sections ‘data collection’ and ‘data analysis’ |
| Data sources/ measurement | 8* | For each variable of interest, give sources of data and details of methods of assessment (measurement). Describe comparability of assessment methods if there is more than one group Methods, section ‘data collection’ |
| Bias | 9 | Describe any efforts to address potential sources of bias Methods, sections ‘data collection’ and ‘data analysis’ |
| Study size | 10 | Explain how the study size was arrived at Methods, section ‘study population and sample’ |
| Quantitative variables | 11 | Explain how quantitative variables were handled in the analyses. If applicable, describe which groupings were chosen and why Methods, section ‘data analysis’ |
| Statistical methods | 12 | (*a*) Describe all statistical methods, including those used to control for confounding Methods, section ‘data analysis’ |
|  |  | (*b*) Describe any methods used to examine subgroups and interactions Methods, section ‘data analysis’ |
|  |  | (*c*) Explain how missing data were addressed N/a, there was no missing data |
|  |  | (*d*) If applicable, describe analytical methods taking account of sampling strategy Methods, section ‘data analysis’ |
|  |  | (*e*) Describe any sensitivity analyses Methods, section ‘data analysis’ |
| Results | | |
| Participants | 13* | (a) Report numbers of individuals at each stage of study—eg numbers potentially eligible, examined for eligibility, confirmed eligible, included in the study, completing follow-up, and analysed Results, paragraphs 1 and 2 |
|  |  | (b) Give reasons for non-participation at each stage N/a |
|  |  | (c) Consider use of a flow diagram We used a table: Table 1 |
| Descriptive data | 14* | (a) Give characteristics of study participants (eg demographic, clinical, social) and information on exposures and potential confounders Results, paragraph 1 and 2 |
|  |  | (b) Indicate number of participants with missing data for each variable of interest There were no missing data |
| Outcome data | 15* | Report numbers of outcome events or summary measures Overview in Fig 1, Table 2 and Table 3, details in supporting information files |
| Main results | 16 | (*a*) Give unadjusted estimates and, if applicable, confounder-adjusted estimates and their precision (eg, 95% confidence interval). Make clear which confounders were adjusted for and why they were included Results, Table 2 and 3 |
|  |  | (*b*) Report category boundaries when continuous variables were categorized N/a |
|  |  | (*c*) If relevant, consider translating estimates of relative risk into absolute risk for a meaningful time period N/a |
| Other analyses | 17 | Report other analyses done—eg analyses of subgroups and interactions, and sensitivity analyses Results, several paragraphs report subgroup analysis (analysis per participant type), an overview can also be found in Table 2 and 3 |
| Discussion | | |
| Key results | 18 | Summarise key results with reference to study objectives Discussion, paragraph 1 |
| Limitations | 19 | Discuss limitations of the study, taking into account sources of potential bias or imprecision. Discuss both direction and magnitude of any potential bias Discussion, section ‘strengths and limitations’ |
| Interpretation | 20 | Give a cautious overall interpretation of results considering objectives, limitations, multiplicity of analyses, results from similar studies, and other relevant evidence Discussion, section ‘interventions to improve the knowledge and perception of leprosy’ |
| Generalisability | 21 | Discuss the generalisability (external validity) of the study results Discussion, section ‘strengths and limitations’ |
| Other information | | |
| Funding | 22 | Give the source of funding and the role of the funders for the present study and, if applicable, for the original study on which the present article is based |

*Give information separately for exposed and unexposed groups.

**Note:** An Explanation and Elaboration article discusses each checklist item and gives methodological background and published examples of transparent reporting. The STROBE checklist is best used in conjunction with this article (freely available on the Web sites of PLoS Medicine at http://www.plosmedicine.org/, Annals of Internal Medicine at http://www.annals.org/, and Epidemiology at http://www.epidem.com/). Information on the STROBE Initiative is available at www.strobe-statement.org.
