# Supplementary material for: Leprosy perceptions and knowledge in endemic districts in India and Indonesia: Differences and commonalities
Source: PLoS Negl Trop Dis. 2021 Jan 21;15(1):e0009031. doi: 10.1371/journal.pntd.0009031 (PMC7853455; doi:10.1371/journal.pntd.0009031)
Supplement: S1 Table — (DOCX) [file pntd.0009031.s002.docx]

Table 1. Number of participants included in the study, per country and per participant group.

| **Participant type** | **Questionnaires^a^** | | **In-depth interviews^b^** | | **Focus group discussions^b^** | |
| --- | --- | --- | --- | --- | --- | --- |
|  | *India* | *Indonesia* | *India* | *Indonesia* | *India* | *Indonesia* |
| Person affected by leprosy | 200 | 238 | 12 | 15 | 16 | 0 |
| Close contact | 211 | 238 | 11 | 18 | 18 | 0 |
| Community member | 556 | 700 | 13 | 8 | 7 | 0 |
| Health care worker | 100 | 101 | 16 | 17 | 19 | 0 |
| Total | 1067 | 1277 | 52 | 58 | 60 | 0 |

^a^ Persons affected were administered the KAP only, while the other participants received the KAP, SDS and EMIC-CSS.
^b^ The qualitative participants are a subset of those in the quantitative sample.
