# Supplementary material for: Leprosy perceptions and knowledge in endemic districts in India and Indonesia: Differences and commonalities
Source: PLoS Negl Trop Dis. 2021 Jan 21;15(1):e0009031. doi: 10.1371/journal.pntd.0009031 (PMC7853455; doi:10.1371/journal.pntd.0009031)
Supplement: S2 Table — (DOCX) [file pntd.0009031.s003.docx]

Table 2. An overview of the number of correct responses given per participant group per country, per knowledge question of the KAP measure.

| **Topic** | **Responses given as percentage of participants (n=2344) who gave the correct answer** | | | | | | | | | | |
| --- | --- | --- | --- | --- | --- | --- | --- | --- | --- | --- | --- |
|  |  | % of persons affected | | % of close contacts | | % of community members | | % of health care workers | | % of all groups | |
|  |  | India (n=200) | Indonesia (n=238) | India (n=211) | Indonesia (n=238) | India (n=556) | Indonesia (n=700) | India (n=100) | Indonesia (n=101) | India (n=1067) | Indonesia (n=1277) |
| *Mode of transmission* | (1) By air | 2.0 | 9.2 | 1.4 | 5.0 | 6.7 | 7.6 | 19.0 | 63.4 | 5.9 | 11.8 |
| *Early symptoms* | (2) Skin patches | 52.0 | 56.7 | 43.1 | 42.4 | 47.1 | 49.9 | 68.0 | 87.1 | 49.2 | 52.7 |
|  | (3) Loss of sensation | 46.0 | 27.3 | 24.2 | 11.8 | 13.3 | 4.0 | 68.0 | 83.2 | 26.7 | 16.1 |
| *Cause* | (4) Germs/bacteria | 3.0 | 11.3 | 9.0 | 9.7 | 9.7 | 10.3 | 62.0 | 88.1 | 13.2 | 16.5 |
| *Contagiousness* | (5) Not contagious when on treatment | 39.5 | 4.6 | 39.8 | 7.6 | 42.6 | 38.7 | 59.0 | 8.9 | 43.0 | 24.2 |
| *Disabilities* | (6) Disabilities can be prevented | 57.5 | 49.2 | 50.7 | 51.3 | 76.4 | 43.1 | 86.0 | 93.1 | 68.7 | 49.7 |
| *Treated how* | (7) By medication | 95.0 | 89.9 | 89.6 | 84.9 | 88.3 | 62.1 | 99.0 | 99.0 | 90.8 | 74.5 |
| *Treatability of leprosy* | (8) Can be treated | 96.0 | 92.9 | 88.6 | 88.2 | 92.4 | 66.9 | 99.0 | 100.0 | 93.0 | 78.3 |
