# Supplementary material for: Leprosy perceptions and knowledge in endemic districts in India and Indonesia: Differences and commonalities
Source: PLoS Negl Trop Dis. 2021 Jan 21;15(1):e0009031. doi: 10.1371/journal.pntd.0009031 (PMC7853455; doi:10.1371/journal.pntd.0009031)
Supplement: S1 Fig — The percentage of participants in India and Indonesia, indicating negative attitudes on the EMIC-CSS and SDS. The percentages are displayed as percentage of participants who answered “yes” (EMIC-CSS) or “definitely not willing” or “probably not willing” (SDS) on each question, out of all participants. (DOCX) [file pntd.0009031.s004.docx]

Fig 1. Percentage of participants in India and Indonesia, indicating negative attitudes on the EMIC-CSS. The percentages are displayed as percentage of participants who answered “yes” on each question, out of all participants.

Fig 2. Percentage of participants in India and Indonesia, indicating negative attitudes on the SDS. The percentages are displayed as percentage of participants who answered “definitely not willing” or “probably not willing” on each question, out of all participants.
