# Supplementary material for: Leprosy perceptions and knowledge in endemic districts in India and Indonesia: Differences and commonalities
Source: PLoS Negl Trop Dis. 2021 Jan 21;15(1):e0009031. doi: 10.1371/journal.pntd.0009031 (PMC7853455; doi:10.1371/journal.pntd.0009031)
Supplement: S1 Text — Regression models for the correlations between level of knowledge about leprosy (KAP measure), community stigma (EMIC-CSS), social distance (SDS) and the other variables in the dataset. (DOCX) [file pntd.0009031.s005.docx]

**Supporting information file**
Correlations between level of knowledge about leprosy (KAP measure), community stigma (EMIC-CSS), social distance (SDS) and the other variables in the dataset.

**Correlations between level of knowledge (KAP measure, in the presence of incorrect answers) about leprosy and the other variables in the dataset

Model using the whole dataset (KAP)**

|  | Regression coefficient | Standard error | p-value |
| --- | --- | --- | --- |
| *(Constant)* | *3.636* | *.071* | *.000* |
| Index patient* | .948 | 1.071 | .376 |
| Close contact* | -.157 | .093 | .092 |
| Health worker* | 2.252 | .121 | .000 |
| Not completed any (formal) education | -.687 | .096 | .000 |
| Completed primary education | -.426 | .090 | .000 |
| Knows someone affected by leprosy | .499 | .079 | .000 |
| From district Pamekasan (Indonesia) | -.657 | .085 | .000 |
| From district Pasuruan (Indonesia) | -.465 | .089 | .000 |

* Included in the model to control for confounding. ‘Community members’ are the reference category.
R-squared = 0.286 **Model for persons affected by leprosy (KAP)**

|  | Regression coefficient | Standard error | p-value |
| --- | --- | --- | --- |
| *(Constant)* | *4.903* | *.255* | *.000* |
| Age | -.010 | .004 | .020 |
| Gender (women) | -.388 | .130 | .003 |
| Not completed any (formal) education | -.368 | .141 | .009 |
| From district Pamekasan (Indonesia) | -.603 | .138 | .000 |

R-squared = 0.120

**Model for contacts and community members (KAP)**

|  | Regression coefficient | Standard error | p-value |
| --- | --- | --- | --- |
| *(Constant)* | 3.738 | .074 | .000 |
| Close contact of person affected by leprosy* | -.117 | .095 | .220 |
| Not completed any (formal) education | -.693 | .097 | .000 |
| Completed primary education | -.383 | .091 | .000 |
| Knows someone affected by leprosy | .416 | .085 | .000 |
| From district Pasuruan (Indonesia) | -.640 | .095 | .000 |
| From district Pamekasan (Indonesia) | -.800 | .091 | .000 |

* Included in the model to control for confounding. ‘Community members’ are the reference category.
R-squared = 0.107

**Model for health workers (KAP)**

|  | Regression coefficient | Standard error | p-value |
| --- | --- | --- | --- |
| *(Constant)* | *7.250* | *.340* | *.000* |
| Gender (women) | -.790 | .173 | .000 |
| Knows someone affected by leprosy | .642 | .203 | .002 |
| From district Pamekasan (Indonesia) | -.572 | .264 | .031 |
| From district Fatehpur (India) | -.966 | .233 | .000 |
| From district Chandauli (India) | -.554 | .241 | .023 |

R-squared = 0.237

**Correlations between level of stigma (EMIC-CSS) and the other variables in the dataset

Model using the whole dataset (EMIC-CSS)**

|  | Regression coefficient | Standard error | p-value |
| --- | --- | --- | --- |
| *(Constant)* | *15.858* | *.332* | *.000* |
| Close contact of person affected by leprosy* | -5.090 | .394 | .000 |
| Health worker* | -2.810 | .543 | .000 |
| From district Pamekasan (Indonesia) | 2.079 | .442 | .000 |
| From district Fatehpur (India) | 1.450 | .497 | .004 |
| From district Chandauli (India) | 1.719 | .442 | .000 |

* Included in the model to control for confounding. ‘Community members’ are the reference category.
R-squared = 0.096

**Model for contacts and community members (EMIC-CSS)**

|  | Regression coefficient | Standard error | p-value |
| --- | --- | --- | --- |
| *(Constant)* | *15.815* | *.344* | *.000* |
| Close contact of person affected by leprosy* | -5.085 | .394 | .000 |
| From district Pamekasan (Indonesia) | 2.239 | .465 | .000 |
| From district Fatehpur (India) | 1.430 | .531 | .007 |
| From district Chandauli (India) | 1.723 | .465 | .000 |

* Included in the model to control for confounding. ‘Community members’ are the reference category.
R-squared = 0.103

**Model for health workers (EMIC-CSS)**

|  | Regression coefficient | Standard error | p-value |
| --- | --- | --- | --- |
| *(Constant)* | *19.363* | *2.019* | *.000* |
| Age | -.126 | .049 | .011 |

R-squared = 0.032

**Correlations between level of social distance (SDS) and the other variables in the dataset (this is a non-normal distribution, we conducted bootstrapping to correct for non-normality)**
 **Model using the whole dataset (SDS)**

|  | Regression coefficient | Standard error | p-value |
| --- | --- | --- | --- |
| *(Constant)* | *7.479* | *.541* | *.001* |
| Close contact of person affected by leprosy* | -.818 | .342 | .021 |
| Health worker* | -2.344 | .460 | .001 |
| Gender (women) | .659 | .253 | .008 |
| Completed higher education | -.928 | .343 | .008 |
| Leprosy knowledge (KAP measure score) | -.367 | .085 | .001 |
| From district Pamekasan (Indonesia) | 1.075 | .355 | .001 |
| From district Pasuruan (Indonesia) | 2.682 | .333 | .001 |
| From district Fatehpur (India) | 1.217 | .353 | .001 |

* Included in the model to control for confounding. ‘Community members’ are the reference category.
R-squared = 0.103

**Model for contacts and community members (SDS)**

|  | Regression coefficient | Standard error | p-value |
| --- | --- | --- | --- |
| *(Constant)* | *7.571* | *.585* | *.001* |
| Close contact of person affected by leprosy* | -.827 | .326 | .011 |
| Gender (women) | .699 | .280 | .009 |
| Completed higher education | -.908 | .350 | .011 |
| Leprosy knowledge (KAP measure score) | -.392 | .091 | .001 |
| From district Pasuruan (Indonesia) | 2.713 | .377 | .001 |
| From district Fatehpur (India) | 1.217 | .408 | .004 |
| From district Pamekasan (Indonesia) | .781 | .400 | .048 |

* Included in the model to control for confounding. ‘Community members’ are the reference category.
R-squared = 0.069

**Model for health workers (SDS)**

|  | Regression coefficient | Standard error | p-value |
| --- | --- | --- | --- |
| *(Constant)* | *7.359* | *1.305* | *.001* |
| Age | -.076 | .030 | .010 |

R-squared = 0.033
