# Supplementary material for: Leprosy perceptions and knowledge in endemic districts in India and Indonesia: Differences and commonalities
Source: PLoS Negl Trop Dis. 2021 Jan 21;15(1):e0009031. doi: 10.1371/journal.pntd.0009031 (PMC7853455; doi:10.1371/journal.pntd.0009031)
Supplement: S2 Text — The results are displayed by participant group, as percentage of participants who gave the correct answer. (DOCX) [file pntd.0009031.s006.docx]

**Supporting information file**An overview of the number of correct responses given per participant group per country, per knowledge question of the KAP measure. The results are displayed by participant group, as percentage of participants who gave the correct answer.


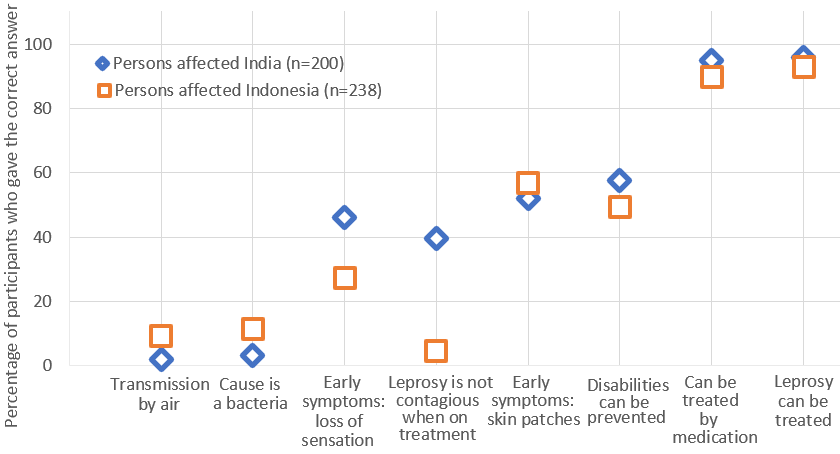
 **Fig 1. The percentage of persons affected by leprosy who gave the correct answer on the KAP measure.**


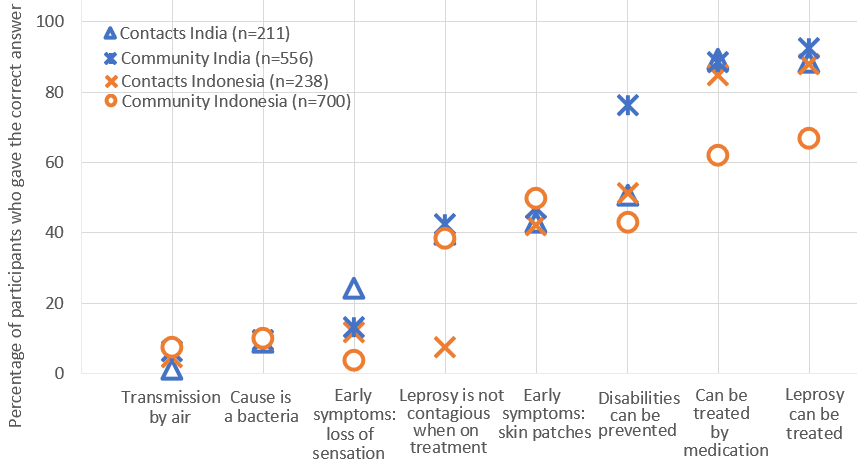
 **Fig 2. The percentage of close contacts and community members who gave the correct answer on the KAP measure.**
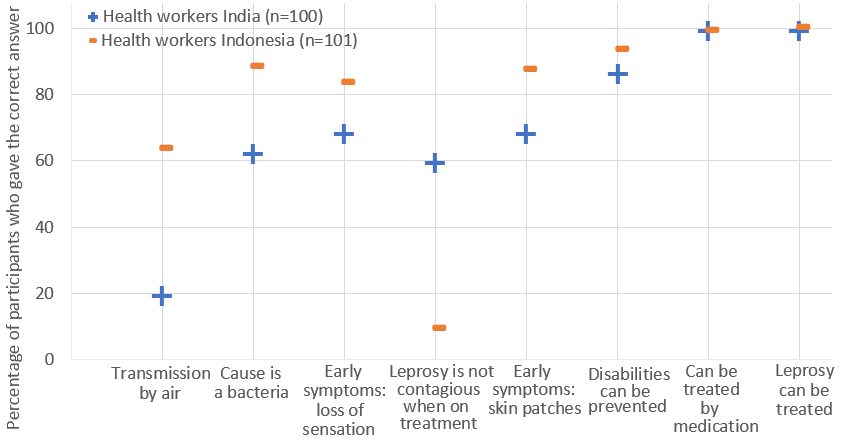
 **Fig 3. The percentage of health workers who gave the correct answer on the KAP measure.**
