# Supplementary material for: Leprosy perceptions and knowledge in endemic districts in India and Indonesia: Differences and commonalities
Source: PLoS Negl Trop Dis. 2021 Jan 21;15(1):e0009031. doi: 10.1371/journal.pntd.0009031 (PMC7853455; doi:10.1371/journal.pntd.0009031)
Supplement: Attachment — Submitted filename: Point-by-point reply_LPSIndiaIndonesia.docx [file pntd.0009031.s008.docx]

**Point-by-point reply document

Manuscript number**: PNTD-D-20-01319 **Manuscript title**: “Leprosy perceptions and knowledge in endemic districts in India and Indonesia: differences and commonalities”
**Deadline for submission of revision**: mid-December

| **Reviewer comment** | **Reaction authors** | **Changes in manuscript (highlighted in yellow)** |
| --- | --- | --- |
| Methodology: the authors should give more details about the focus group (…) the article does not specify the profile of the participants or bring the ideas and insights raised in the group interaction. | The following information about the profile of the focus group participants has been reported: “and in India 60 participants were included in seven focus group discussions (average age 40 years, gender not recorded).” (line 264-265). Supporting information file S1 contains a breakdown of number of participants per participant group (FGD: 16 persons affected, 18 close contacts, 7 community members, 19 health workers). Unfortunately, no further information about the profile of the focus group participants was collected.  Final analysis contained clustering of the data of the in-depth interviews and FGDs. We now indicate per quote whether it was from an in-depth interview or a focus group discussion (FGD).  Since we now noticed that we had only included quotes from the in-depth interviews, we added a quote from a FGD. | Added: line 301: “Both on the KAP measure and during the in-depth interviews and focus group discussions, participants often used multiple explanations and held different beliefs per knowledge topic.…” and line 379 “In the in-depth interviews and focus group discussions, many participants indicated that community members have a negative attitude towards persons affected by leprosy and avoid or exclude persons affected by leprosy.” (since the results describe data from the in-depth interviews and focus group discussions).  In addition, after each quote we added whether the quote was from an in-depth interview or focus group discussion (FGD).  We added the following quote from the FGDs, line 398-403: “…People do not eat with leprosy patient nor touch them. And his living place is also separated. They refuse to visit some places and says don’t touch me, or else I will also have disease. Because of this he becomes sad and suffers from inferiority complex. Sometimes he also tried to commit suicide…” - Community member, gender unknown, India, focus group discussion |
| Authors should include information on how minors' consent was obtained. | This has been added. | The following has been added to methods > ethical considerations line 242-244:  “For minors (participants below 18 years of age), verbal informed consent was obtained from the minor and written informed consent was obtained from one of the minor’s legal representatives, e.g. a parent or guardian” |
| Gaps and problems statements/hypothesis / are absent. The gap should be indicated since there are many researches related to this study many where. The objectives of the study were clearly articulated. | We aimed to indicate the knowledge gap in the final paragraph of our introduction. We have tried to make this clearer. | The following (in yellow) has been added to line 102:  “Although knowledge, attitudes and practices regarding leprosy have been studied extensively and different determinants of knowledge and community stigma have been identified, we lack consensus about determinants of stigma and insight into how we can change negative perceptions and stigmatising local beliefs about leprosy.  Insight in the dynamics, differences and commonalities in knowledge, attitudes and practices between leprosy endemic countries can help us to better target interventions to improve the knowledge and perception of leprosy, and thus to reduce stigma. This study aims to examine the differences and commonalities in and determinants of knowledge, attitudes and practices regarding leprosy in leprosy endemic districts in India and Indonesia.” |
| Methods, eligibility criteria: Previous research in which I my self involved in Ethiopia shows as you move appart from the neigbourhood, the negative attitude, knowlegde about the contagious property increase. There is one village created as confining center about 95 years back. Poeple in the vicinity are in a very close contact due to the knowledge that they simply obtained in time. So, you need to validate by looking the difference between the distant and close community. | We thank the reviewer for his/her comment and feedback. We hope we have clarified this in our methods section. We have included close contacts such as household members and neighbours (“close contacts”) and community members. This is also stated on line 125-126 (study population and sample): “Four groups of people were included in the study: (1) persons affected by leprosy; (2) close contacts of persons affected; (3) community members; and (4) health care workers.” And in the eligibility criteria. In the eligibility criteria we have tried to explain that community members refer to ‘more distant’ contacts (not close contacts). We hope this is explained sufficiently in this way. | - |
| Methods, sampling methods: You mean 20 each? (referring to “20 close contacts”, line 154) | This has been revised. | Line 152: “All persons affected included in the study were each asked to name their 20 closest contacts whose names were written down on pieces of paper.” |
| Methods, data collection: Do not you find documents/religious, legal / in both country which prohibite or encourage stigima , knolegde transimition or any situation related to your study as common/different. *(and)* The two countries may have documents to prevent negative views, bring about common knowledge or any other concerns which should support the study. I found the study well triangulated but still if things related to the problems are not abide by legal, or social rules people may not consider it forbidden to disregard leprosy affected people. So, would the authors consider this? | We thank the reviewer for his or her comment. Although interesting, the analysis of religious scripts and legal documents was beyond the scope of our study. We therefore did not include this. We hope the reviewer will understand this. We have also emphasized the importance of exploring religion in a recommendation in our discussion, line 550-552: “In addition, we think it would be helpful to explore the influence of religion better, not just taking beliefs, but also level of religious faith and dedication into account.”  We have changed our conclusion based on the reviewers’ comments. | Line 622-623: “We also recommend that future studies ~~should also~~ explore the role of religion, religiosity and area of residence in local beliefs.” |
| The sample size seem representative though there were discrepancy between two countries in sample selection . This should be justified. *(and)* Why the selection of contacts from individuals in India was different from contacts in Indonesia. | We have added a line to make this clear, thank you.  This is also mentioned as study limitation. (“Another limitation of this study is the difference in sampling methods of close contact selection in India and Indonesia. Close contacts in India were selected by convenience sampling, while close contacts in Indonesia were selected by random sampling. Despite differences in recruitment methods, the patterns of results showed parallels”) | The following was added to line 150-153 (methods): “In India, close contacts were selected by convenience sampling. We realized in hindsight that convenience sampling was not the best approach and therefore applied random sampling of close contacts when initiating the data collection in Indonesia. In Indonesia, all persons affected included in the study were each asked to name their 20 closest contacts whose names were written down on pieces of paper. (…)” |
| I trusted the authors and PLOS NEGLECTED TROPICAL DISEASES that I did not find attachment of Ethical clearance Certificate. I believe it should be there. Thank you. | We thank the reviewer for this comment. If required by PLOS NTD, we can of course share the ethical clearance certificate. | - |
| Remove figure 1. It contains too much data making it impossible to interpret the information (line 297). | We thank the reviewer for his/her comment. We have removed figure 1 from the manuscript. The information is now only available in supporting information file S2 and S5 (per participant group). | We have adjusted the supporting information captions, so that no reference to Figure 1 is made.   In addition, we have added “An overview of the number of correct responses given per participant group per country, per knowledge question of the KAP measure can be found as supporting information files (S2 and S5).” to the results section (line 273-275). These supporting information files refer to a breakdown of Figure 1, so readers who are interested can look this up in the supporting information file. We agree with the reviewer that Figure 1 contains too much information. |
| The analysis presented match the analysis plan that the KAP (variables to be investigated were presented with the determinants). The reviewer found  the results were clearly and completely presented from the perspectives of the objectives, and they were supported with figures and tables. Still there are areas which need validation regarding commonalities and differences in cases of neighborhood and distant communities' attitude. To be crosschecked... because it seemed generalization without clear consideration. I my self have a brother who is affected by leprosy. People in the distant area consider him as a unique and disregard from any participation. However the closer communities do not. It was due to that I conducted a communication research which mainly focuses on discriminatory perception, attitude and discourse.  So, Completely I  disagree that the neighbors  have negative attitude towards leprosy affected people.  Except this, every thing here is very fine. | We thank the reviewer for this comment. Indeed, close contacts might respond differently than community members. Therefore we first analysed their data separately. This is reflected in, for example, Table 2 (both KAP, EMIC-CSS and SDS scores of close contacts, community members). This indeed shows a difference between contacts and community members, contacts for example have a lower EMIC-CSS and SDS score (Table 2).  In spite of these differences we decided to pool data of close contacts and community members in the multivariate regression models. Separate models for health workers were made because we considered them a more heterogenous group and a different group than the general population in terms of having completed (higher) education, knowledge (training) about leprosy and occupation. Separate models for persons affected were made because of their personal experience with leprosy and because they likely received a briefing or information about their condition. Close contacts and community members were pooled, because we considered them both to be of the ‘general population’ when looking at determinants for the KAP, EMIC-CSS and SDS scores. We explored separate models for contacts and community members also (these were similar), but decided not to include these in the manuscript. This was done because we wanted to use the information for interventions to improve the knowledge and perception of leprosy (and thus reduce stigma). These interventions would be similar for close contacts and community members. In addition, not all close contacts were aware that they were a close contact of a person affected by leprosy.   We hypothesized that participant type correlated with dependent and independent variables in the model. To control for confounding of participant type, and to ensure that the effects of participant type would be removed from the final results, we decided to always keep all participant types in the model, when analysing the whole dataset and when making models for close contacts and community members.  We kindly refer the reviewer to our methods section, line 222-232, for more details.  We will explore the differences between close contacts and community in more detail in the next phase of the PEP++ project, of which the perception studies are part. | - |
| Knowing that health professionals are those who promote health education among affected individuals, contacts, and the community, it is interesting to note that the lack of knowledge about a particular topic among professionals is reflected in erroneous knowledge among affected individuals, contacts, and community members. In the same way that adequate knowledge on a given topic enables the professional to pass on correct information, as can be seen in the results shown in the S5 Figure. I suggest that the authors address these results in the discussion. | We thank the reviewer for this helpful comment. This has been added. | We have added to our discussion (line 563-570): “Interestingly, the topics on which health workers lacked knowledge (e.g. mode of transmission and contagiousness after treatment) and had adequate knowledge (e.g. treatment) was reflected in the knowledge about leprosy among the other participant groups. This likely shows that adequate knowledge about a particular topic among health workers enables them to pass on correct information to persons affected, contacts and community members. In addition, we believe it reflects the messages that have been used in past government education campaigns. These findings underline the importance of ensuring that health workers have correct knowledge about leprosy.” |
| Results, Differences and commonalities in leprosy knowledge and beliefs: Does your data suggest cause of diferences in knoldege, attitude andperception or belife. If so, elaborating a little bit about it may make the information very complete. I do not mean that it is not enough. *(and)* I little bit mentioned in the main manuscript the presence of vagueness of data presentation to show how much they clearly indicate  saying "Does your data suggest cause of differences in knowledge, attitude and perception or belief. If so, elaborating a little bit about it may make the information very complete" to increase clarity. In fact all the concluding points were emanating from the data presented | We thank the reviewer for his/her comment. Our results are divided in several sections: 1. Demographic information 2. Differences and commonalities in knowledge, attitudes and practices regarding leprosy  2.1 Differences and commonalities in leprosy knowledge and beliefs 2.2 Differences and commonalities in leprosy-related stigma 3. Determinants of leprosy knowledge and leprosy-related stigma  3.1 Determinants of knowledge of leprosy 3.2 Determinants of leprosy-related stigma  We have added the numbering to make it more clear where what information can be found. The cause of the differences in knowledge can be found in the ‘determinants’ section. For example: “Multivariate analysis showed that living in Chandauli, Fatehpur or Pamekasan district was associated with higher levels of stigma towards persons affected by leprosy (Table 3). This model explained 10% of the variability of stigma.” (line 440-442).   In addition, we believe some of the differences could potentially be explained by religion. We have mentioned this in our discussion also, on line 484-486 “Some studies have suggested that these beliefs are influenced by religious beliefs and religious teachings about leprosy [13,19,20,38]. We hypothesize that the local beliefs in the present study have to some extent also been influenced by religion and religious practices. …” | - |
| “We found that 12% of the participants in India and 10% of the participants in Indonesia had adequate knowledge of leprosy” (line 293-294) remark reviewer: Adequecy standard you set as operational defenition may be vegue. What exactly adequete indicates? | We thank the reviewer for his/her comments. The definitions are stated in the methods section, line 181-184: “We defined ‘poor knowledge’ as a score of two or less out of eight on the KAP measure (≤25% correct), ‘moderate knowledge’ as a score between two and six (25-75% correct) and ‘adequate knowledge’ as a score of six or more (≥75% correct).” | - |
| Results, line 372-380: reviewer comment: Do not you think we have to know why of these? As it makes intervention plausable. | Thank you, this has been revised and clarified. | We have added “An overview of the number of correct responses given per participant group per country, per knowledge question of the KAP measure can be found as supporting information files (S2 and S5).” to the results section (line 273-275). These supporting information files refer to a breakdown of Figure 1, so readers who are interested can look this up in the supporting information file. We agree with the reviewer that Figure 1 contains too much information. |
| I suggest the authors eliminate unnecessary spaces between paragraphs during the text. Line 348 - insert the numbering for the topic. | We thank the reviewer. This has been revised, not using track changes (for clarity). We hope this is sufficient and welcome other suggestions/edits. | The numbering has been added. |
| Confirm whether the number of individuals interviewed was 110 or 100. | We aimed to include at least 100 participants per target group (also stated in methods > study population and sampling, line 126). A total of 110 participants (52 in India, 58 in Indonesia) were included/interviewed in-depth (stated on line 260). | - |
| There should be theoretical foundation of the study(I have not noticed ). *(and)* Theoretical foundations and gap to be field were not well explained. Differences were overlooked. *(and)* In the introduction of the text much is said about stigma and the other elements that will also be addressed in the study are not highlighted. I suggest that in addition to stigma, also mention more about social determinants and knowledge | We thank the reviewer for his/her comment. Our study was cross-sectional and aimed to examine the differences and commonalities in and determinants of knowledge, attitudes and stigma regarding leprosy. The ‘perception study toolkit’ we used was developed with the sitmga intervention framework (described [here](https://bmcmedicine.biomedcentral.com/articles/10.1186/s12916-018-1245-x)) in mind. In addition, we have used information from other studies (literature) to guide our research. This is in part explained in our introduction, line 87-95: “There are many factors that contribute to the stigma of leprosy, including fear of transmission and contagion, the visible manifestations such as deformity and disability in persons affected and religious and cultural beliefs regarding causes and treatment of leprosy [12,13]. Knowledge about leprosy plays a crucial role in stigma [14–18]. Local (mis)beliefs, such as the beliefs that all leprosy patients end up with disabilities, that leprosy is not curable or results in death or that imply that the person affected has done wrong and brought the disease upon himself all contribute to stigma [13,14,18–23]. Research showed that personal characteristics such as gender [15,16,22,24], occupation [16,22,24–26], years of education [15–17,22,25], age [15,25,27,28] and living area [15,24,27,29,30] are associated with community stigma against persons affected by leprosy.”  In addition, we have used validated tools that have also been used in previous studies, to collect our data.   In addition, differences are mostly highlighted in our qualitative data, especially in line 311-343. (“…In addition, some participants in Indonesia believed there were two types of leprosy: ‘lepra’, the skin condition and ‘*kusta’*, a more severe and feared form with visible impairments. Many participants in India thought leprosy transmits by touch and linked this to ‘untouchability’ and sometimes to being religiously unclean”) | - |
| The study aims to examine the differences and commonalities in the determinants for knowledge, attitudes, and practices related to leprosy (lines 102-104). However, I believe that the study goes further by bringing results that also address stigma and prejudice, so I suggest that the authors review the objective of the study. | We thank the reviewer for this helpful comment. We have currently mentioned ‘attitudes’ in our aim, as this is a proxy for stigma and prejudice. We have now added ‘fears’ to our aim, since this is something we explored, but did not mention in our aim previously. | We have adjusted our aim slightly: “This study aimed to examine the differences and commonalities in and determinants of knowledge, attitudes, ~~and~~ practices and fears regarding leprosy in endemic districts in India and Indonesia.” (In the abstract and introduction). |
| Request from PLOS NTD: “We noticed that you used "unpublished" data in the manuscript, we do not allow these references. The PLOS data access policy requires that all data be either published with the manuscript or made available in a publicly accessible database. Please amend the supplementary material to include the referenced data or remove the references. Please make a note of this change in your response to reviewers” | This has been revised. | On line 201-202 we initially wrote “The KAP measure has been used in several (unpublished) leprosy studies in Nepal, India, Indonesia and Brazil between 2012 and 2017.” We have now removed the word “unpublished”.   We have added the references of the (published) studies in which the KAP was used.   Line 201-202 now reads: “The KAP measure has been used in several leprosy studies in Nepal, India, Indonesia and Brazil between 2012 and 2017 [18,33–35].” |
